# Longitudinal changes in COVID-19 vaccination intent among South African adults: evidence from the NIDS-CRAM panel survey, February to May 2021

**DOI:** 10.1186/s12889-022-12826-5

**Published:** 2022-03-02

**Authors:** Ronelle Burger, Timothy Köhler, Aleksandra M. Golos, Alison M. Buttenheim, René English, Michele Tameris, Brendan Maughan-Brown

**Affiliations:** 1 Research on Socio-Economic Policy, Department of Economics, Stellenbosch University, Stellenbosch, South Africa; 2 Development Policy Research Unit, School of Economics, University of Cape Town, Cape Town, South Africa; 3 Department of Family and Community Health, University of Pennsylvania School of Nursing, 416 Fagin Hall, 418 Curie Blvd, Philadelphia, PA 19104 USA; 4 Division of Health Systems and Public Health. Department of Global Health, Faculty of Medicine and Health Sciences, Stellenbosch University, Stellenbosch, South Africa; 5 South African Tuberculosis Vaccine Initiative, Department of Pathology, Institute of Infectious Diseases and Molecular Medicine, University of Cape Town, Cape Town, South Africa; 6 Southern Africa Labour and Development Research Unit, School of Economics, University of Cape Town, Cape Town, South Africa

**Keywords:** COVID-19 vaccine, Vaccine acceptance, Vaccine hesitancy, South Africa, Panel survey

## Abstract

**Background:**

COVID-19 vaccine hesitancy has threatened the ability of many countries worldwide to contain the pandemic. Given the severe impact of the pandemic in South Africa and disruptions to the roll-out of the vaccine in early 2021, slower-than-expected uptake is a pressing public health challenge in the country. We examined longitudinal changes in COVID-19 vaccination intent among South African adults, as well as determinants of intent to receive a vaccine.

**Methods:**

We used longitudinal data from Wave 4 (February/March 2021) and Wave 5 (April/May 2021) of the National Income Dynamics Study: Coronavirus Rapid Mobile Survey (NIDS-CRAM), a national and broadly representative panel survey of adults in South Africa. We conducted cross-sectional analyses on aggregate and between-group variation in vaccination intent, examined individual-level changes between waves, and modeled demographic predictors of intent.

**Results:**

We analysed data for 5629 (Wave 4; 48% male, mean age 41.5 years) and 5862 (Wave 5; 48% male, mean age 41.6 years) respondents. Willingness to get a COVID-19 vaccine significantly increased from 70.8% (95% CI: 68.5–73.1) in Wave 4 to 76.1% (95% CI: 74.2–77.8) in Wave 5. Individual-level analyses indicated that only 6.6% of respondents remained strongly hesitant between survey waves. Although respondents aged 18–24 years were 8.5 percentage points more likely to report hesitancy, hesitant respondents in this group were 5.6 percentage points more likely to change their minds by Wave 5. Concerns about rushed testing and safety of the vaccines were frequent and strongly-held reasons for hesitancy.

**Conclusions:**

Willingness to receive a COVID-19 vaccine has increased among adults in South Africa, and those who were entrenched in their reluctance make up a small proportion of the country’s population. Younger adults, those in formal housing, and those who trusted COVID-19 information on social media were more likely to be hesitant. Given that stated vaccination intent may not translate into behaviour, our finding that three-quarters of the population were willing to accept the vaccine may reflect an upper bound. Vaccination promotion campaigns should continue to frame vaccine acceptance as the norm and tailor strategies to different demographic groups.

**Supplementary Information:**

The online version contains supplementary material available at 10.1186/s12889-022-12826-5.

## Background

Human behaviour is a key determinant of the effectiveness of vaccines in controlling the COVID-19 pandemic. For vaccines to control the spread of COVID-19, a high percentage of the population must be vaccinated [1, 2]. However, studies from many countries worldwide find that a large proportion of individuals report COVID-19 vaccine hesitancy, or concerns or reluctance about the vaccines that affect intention to seek or accept a vaccine once available [3]. Vaccine hesitancy itself is a complex phenomenon, influenced by a range of cognitive, psychological, socioeconomic, cultural, social, and environmental factors [3, 4]. Large regional differences in vaccine hesitancy underscore both the complexity of the problem and the importance of detailed country-level analyses. Within each setting, it is important to understand the degree of vaccine hesitancy, its drivers, and its evolution over time.

The concern about COVID-19 vaccine hesitancy in South Africa is motivated by the pandemic’s severe impact on the country. In June and July 2021, in the midst of a third wave of infections, South Africa was one of twenty countries worldwide most impacted by the COVID-19 pandemic, ranking sixth among this group for case fatality ratio [5, 6]. This study examines COVID-19 vaccine hesitancy in South Africa amidst the heightened uncertainty, anxiety, and distrust due to the pandemic, as well as the slow emergence of a feasible strategy to vaccinate the country’s adult population. Data collection took place between February 2 and May 11, 2021, in the early phase of vaccine rollout. At this time, only health workers were eligible to receive a vaccine. Registration for Phase II (elderly individuals and essential workers) opened on April 16, but vaccinations for these groups had not yet started. As in many other middle-income countries, the government in South Africa initially struggled to procure enough vaccines and only managed to resolve this supply shortage by mid-August. Against this backdrop, this study assesses the stability and predictors of vaccination intent, with the aim of providing insights that can guide interventions to increase vaccine demand. The lessons learned from this study are relevant for low- and middle-income countries that may experience similar challenges to their vaccination rollout programmes.

## Methods

### Survey design

We analyse data from the latest two waves of the National Income Dynamics Study: Coronavirus Rapid Mobile Survey (NIDS-CRAM), a national and broadly representative longitudinal survey of adults in South Africa, to determine the proportion of adults who reported willingness or hesitancy to receive a COVID-19 vaccine. The objective of the NIDS-CRAM survey was to create a nationally representative, rapid, and longitudinal dataset that could inform evidence-based policy-making during the COVID-19 pandemic. The survey instrument, which is available online, includes a range of questions pertaining to employment, welfare, hunger, government assistance, and COVID-19-related beliefs [7]. The sample was drawn from a subsample of adult respondents from the latest wave of the National Income Dynamics Study (NIDS), a nationally representative longitudinal survey of initially over 28,000 South African adults that tracked social and economic outcomes from 2008 to 2017. Although the original NIDS longitudinal study was administered in person, the NIDS-CRAM study was administered telephonically. Wave 1, which surveyed 7073 adults, was conducted in May and June 2020, shortly after the onset of South Africa’s national lockdown at the end of March. Due to attrition between Waves 1 and 2 (approximately 19%), the sample in Wave 3 was replenished with a top-up sample of 1084 respondents. Our analysis uses data from the latest two waves of NIDS-CRAM that include data on vaccination intent, Waves 4 (conducted in February/March 2021) and 5 (April/May 2021).

### Study setting

**Fig. 1:**
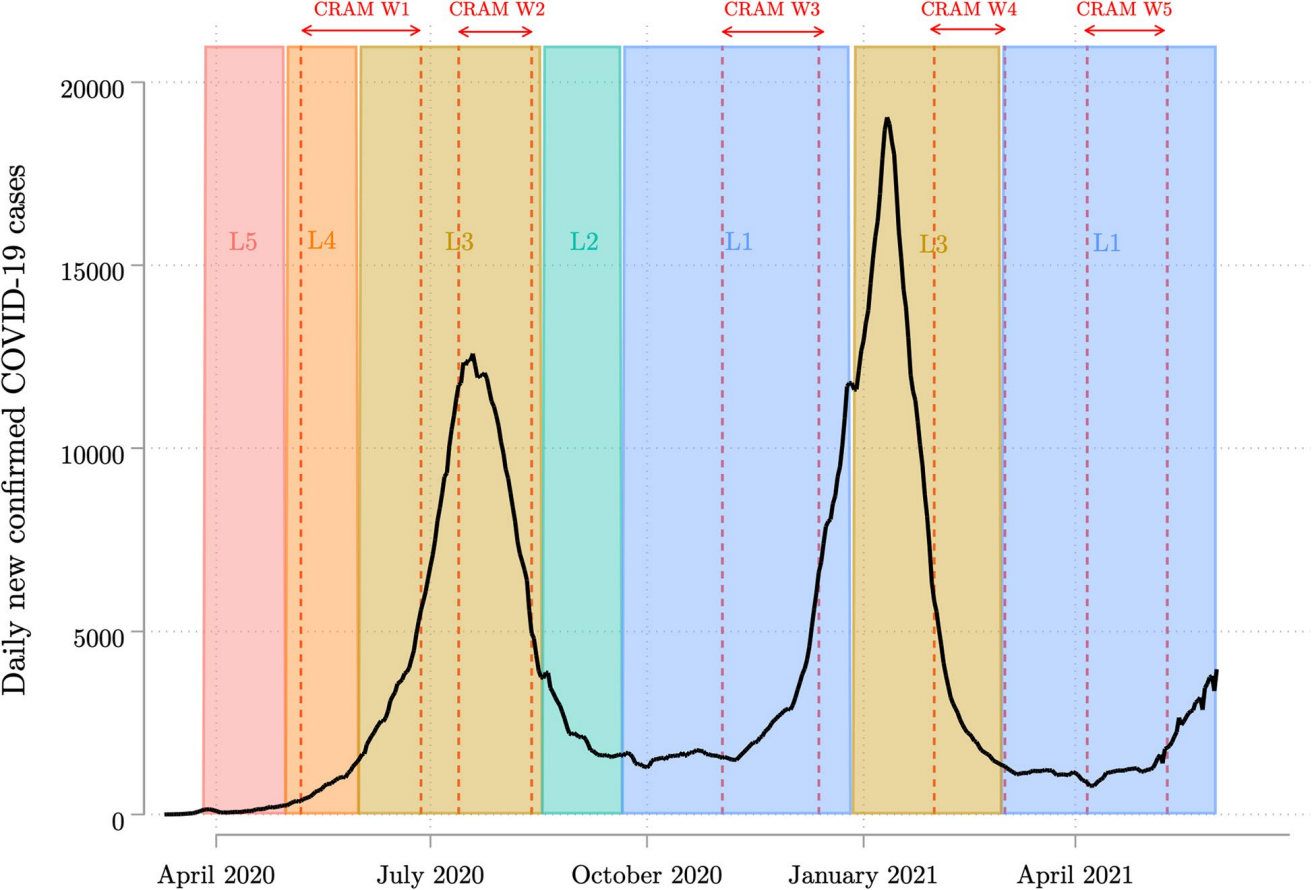
Timing of the NIDS-CRAM waves with respect to COVID-19 cases and lockdown levels in South Africa. Authors’ own arrangement. Source of COVID-19 case data: Our World in Data [8]. Solid line represents 7-day rolling average of daily new confirmed COVID-19 cases. L = lockdown level

**Fig. 2:**
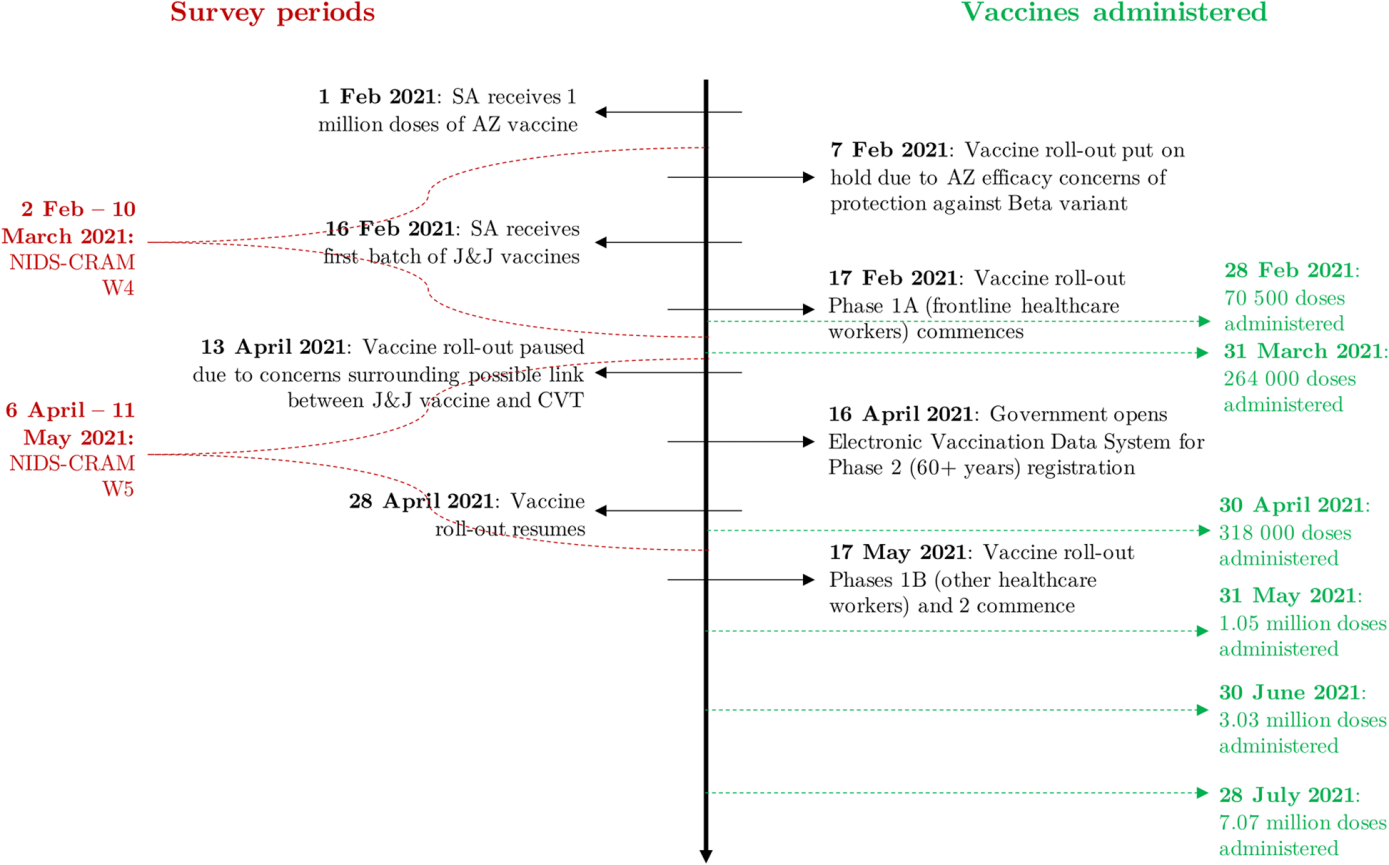
Timeline of NIDS-CRAM survey dates, vaccine-related events, and administered doses in South Africa. Authors’ own arrangement. Source of vaccine dose data: Our World in Data [8]

For context, Fig. 1 illustrates the timing of the five NIDS-CRAM waves with respect to daily new confirmed COVID-19 cases and lockdown alert levels (with level 5 being the most stringent and 1 the most lenient). Important vaccine-related developments that coincided with Waves 4 (February 2 to March 10, 2021) and 5 (April 6 to May 11, 2021) are indicated in Fig. 2 alongside the number of vaccine doses administered. Of note, the vaccination programme was placed on hold twice during this period. On February 7, it was announced that the Oxford-AstraZeneca vaccine had limited efficacy against the dominant Beta variant, and the country’s doses were sold to other African Union member countries [9]. From April 13 to 28, administration of the Johnson & Johnson vaccine was temporarily suspended in light of concerns about its possible association with cerebral venous thrombosis [10]. The total amount of administered vaccinations surpassed 125,000 by the end of Wave 4 and 415,000 by the end of Wave 5 [11]. The surveys also preceded the widespread vaccination of individuals aged 60 years or older, which began on May 17 [12].

### Outcome variables

Vaccine-related survey questions are provided in Additional file 1. Our main outcome variable was COVID-19 vaccination intent. In both waves of the survey, we asked respondents to indicate the extent of their agreement with the statement “if a vaccine for COVID-19 were available, I would get it”, with response options being “strongly agree”, “somewhat agree”, “somewhat disagree”, “strongly disagree”, and “I don’t know”. In Wave 5, respondents were first asked if they had already been vaccinated, and they skipped the rest of the vaccine module if so. Vaccine willingness was defined to include respondents who “strongly” or “somewhat” agreed with the statement, and vaccine hesitancy was defined to include those who “strongly” or “somewhat” disagreed, as well as those who said that they did not know.

To better understand motivations, vaccine-hesitant respondents in Wave 5 were asked whether they thought the vaccine was unsafe or could harm them. If they responded “yes”, they were asked how convinced they were of this, with response options being “a little”, “somewhat”, or “very” convinced. Finally, respondents were asked the open-ended question, “Why do you believe the vaccine is unsafe or harmful?” Interviewers were provided with eight categories (corresponding to findings from exploratory work on vaccine beliefs) for coding responses, but they were instructed not to read out these categories. Responses were coded to existing categories if applicable, or were captured as free text by the interviewer and then later categorised by a research psychologist using thematic analysis.

### Covariates

We drew on a wide range of information about respondents’ demographic, ethnic, social, and economic characteristics, collected in the NIDS-CRAM as well as from their records in previous NIDS waves. We included variables capturing settlement type, province, age (18–24, 25–59, 60 and older), gender, population group (black African, Coloured, White, and Asian/Indian), language spoken at home, and self-reported religious affiliation. We used two questions regarding COVID-19 risk beliefs in our analysis: a question asking whether respondents thought they were likely to get the Coronavirus, and a question asking whether they thought they could avoid getting the virus. Regarding medical risk factors, we included biometric data on body mass index and blood pressure from two repeated measurements from NIDS Wave 5 (2017). We also included responses to the question, “Do you have any of these chronic conditions (you don’t have to tell us which one): HIV, TB, lung condition, heart condition or diabetes?” from NIDS-CRAM Wave 1. We also included an open-ended question from NIDS-CRAM Wave 1 asking respondents where they get information about COVID-19 that they trust. Finally, to examine variation in vaccine hesitancy by income or wealth, we relied on several measures of socioeconomic status. Due to concerns about reliability of and bias in a household income variable captured in the survey, we generated a “deprivation and poverty” household asset index as a proxy to capture differences in socioeconomic status (see Additional file 2 for more details). Additionally, we used respondents’ report of recent hunger in the household and receipt of a means-tested state cash transfer (social grant) as proxies for socioeconomic status.

### Statistical analysis

For each wave, we conducted cross-sectional analyses on aggregate and between-group variation in vaccine hesitancy. Transition matrices were used to examine individual-level changes in vaccine willingness between NIDS-CRAM Wave 4 and Wave 5. We also employed bivariate descriptive analyses as well as a multivariable linear probability model to examine the correlations between vaccination intent and a large number of demographic characteristics and individual attributes. Estimates were weighted using the relevant sampling weights, drawn from the 2017 NIDS survey, to account for the complex survey design and to adjust for non-random non-response and attrition [13, 14]. In our regression analysis of predictors of vaccine hesitancy and changes in vaccine hesitancy across the two survey waves, we included age, gender, population group, language spoken at home, religious affiliation, beliefs about COVID-19, comorbidities, and trusted information sources. Our analyses employed a 5% significance level to assess the precision of estimates.

## Results

### Sample characteristics

Wave 4 of NIDS-CRAM was conducted with an initial sample of 5629 respondents (weighted to be 48% male, mean age = 41.48 years), and Wave 5 was conducted with an initial sample of 5862 (weighted to be 48% male, mean age = 41.57 years). Compared to Wave 1 (May and June 2020), Wave 4 had 31% attrition and Wave 5 had 28% attrition. Table 1 displays relevant descriptive characteristics of the respondents in each sample.

**Table 1:** Sample characteristics, NIDS-CRAM Waves 4 and 5

	Wave 4		Wave 5		Wave 4/5, Balanced Panel	
	n	Weighted %	n	Weighted %	n	Weighted %
**Gender**						
Male	2156	48	2248	48	1875	49
Female	3473	52	3614	52	3074	51
**Age**						
18–24	864	15	846	14	717	15
25–59	3920	70	4136	71	3494	71
60+	845	15	880	15	738	14
**Racial population group**						
African/ Black	4896	79	5072	79	4298	70
Coloured	440	10	490	10	395	10
Indian/ Asian	43	2	46	2	39	2
White	250	9	254	9	217	9
**Highest education**						
Up to primary	1007	14	1037	14	879	14
Up to secondary	2138	36	2214	36	1870	36
Completed secondary	1293	22	1338	22	1149	22
Tertiary	1147	28	1224	28	1014	28
**Residential area**						
Rural	1684	24	1708	24	1464	24
Urban	3693	76	3855	76	3253	76
**Chronic conditions**						
No	3673	81	3862	81	3215	81
Yes	1103	19	1116	19	974	19
**Per capita household income quintile**						
1	1261	21	1459	20	1236	21
2	1355	19	1147	22	977	19
3	1150	20	1181	20	1015	20
4	876	21	1075	18	897	21
5	642	18	605	20	503	19
**Total**	**5629**	**100**	**5862**	**100**	**4949**	**100**

Note: Authors’ own calculations. Source: NIDS-CRAM Waves 4 and 5. Relevant estimates weighted using sampling weights

### Intent to receive a COVID-19 vaccine

**Fig. 3:**
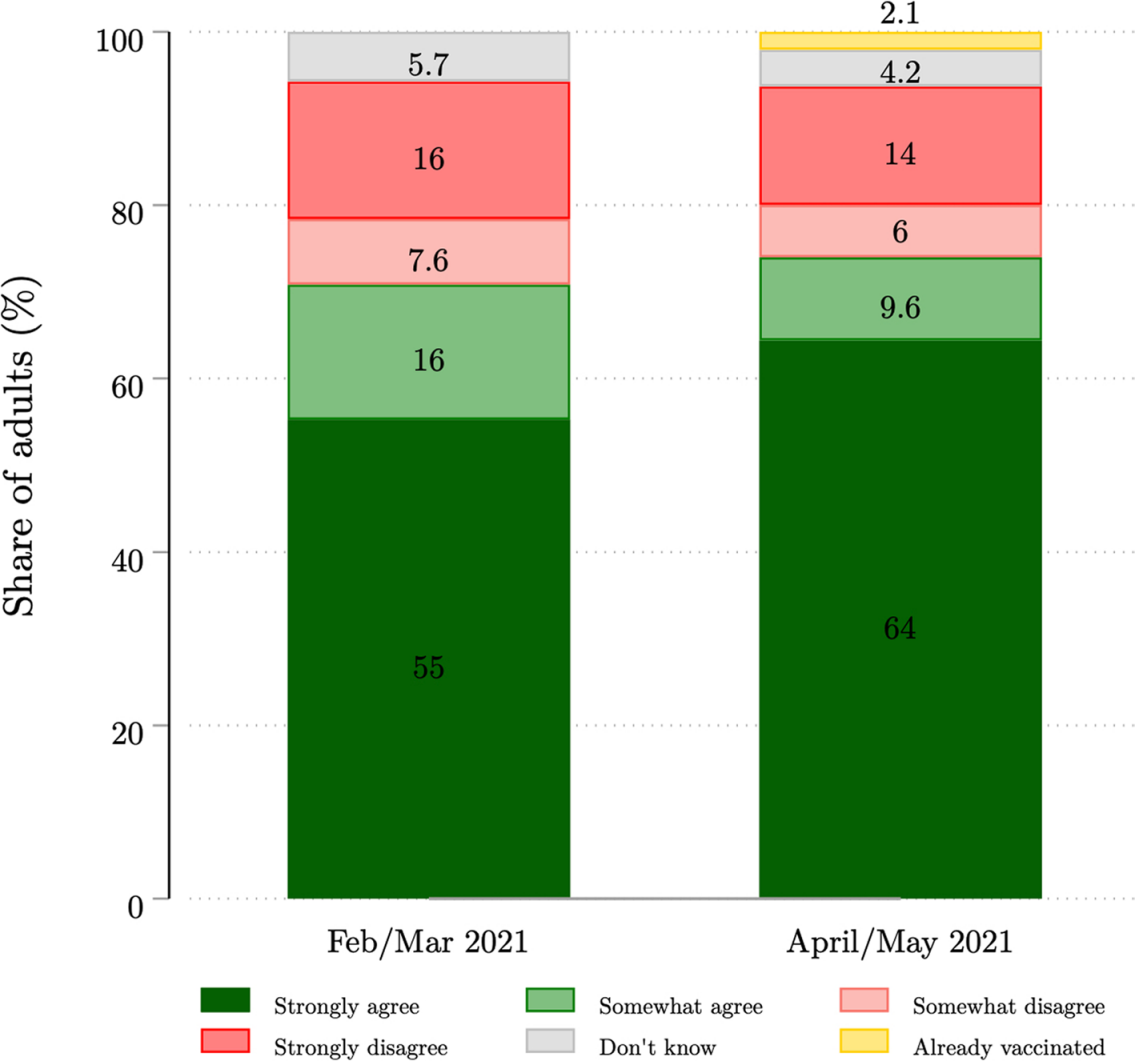
Self-reported intent to receive a COVID-19 vaccine, NIDS-CRAM Waves 4 and 5. Authors’ own calculations. Source: NIDS-CRAM Waves 4 and 5. Relevant estimates weighted using sampling weights

We found a substantial but declining degree of COVID-19 vaccine hesitancy among South African adults, as illustrated in Fig. 3. In Wave 4 (February/March 2021), 29.2% disagreed with the statement that they would get a vaccine if one were available to them (15.9% “strongly disagree”, 7.6% “somewhat disagree”, 5.7% “don’t know”). Most of the 70.8% of adults who were willing to get a vaccine felt strongly about this (55.2% “strongly agree”, 15.6% “somewhat agree”). In Wave 5 (April/May 2021), the proportion unwilling to get a vaccine fell to 24.0% (13.7% “strongly disagree”, 6.1% “somewhat disagree”, 4.2% “don’t know”), and the proportion willing rose to 74.0% (64.4% “strongly agree”, 9.6% “somewhat agree”). 2.1% of the Wave 5 sample had already been vaccinated. Including the already vaccinated among the willing, this observed increase in vaccine acceptance from 70.8% (95% CI: 68.5–73.1) to 76.1% (95% CI = 74.2–77.8) is statistically significant. Of note, the greatest degree of change between the two waves was seen in the “strongly agree” category, increasing by 9.2 percentage points over this short period.

### Changes in intent to receive a COVID-19 vaccine between waves 4 and 5

The transition matrices in Tables 2 and 3 display changes in intent to get a COVID-19 vaccine between Waves 4 and 5. Data were analysed for those who were interviewed in both waves (*n* = 4931), and therefore represent individual-level changes. The use of appropriate panel sampling weights account for between-wave attrition and the representativeness of the sample. Our results demonstrate that willingness to get a vaccine was more stable than reluctance to get a vaccine. 86.96% of respondents who indicated willingness in Wave 4 did so again in Wave 5, compared to 31.34% of those who indicated hesitancy. Moreover, only 42.95% of respondents who strongly disagreed with getting a vaccine in Wave 4 strongly disagreed in Wave 5, whereas 40.06% shifted to strongly or somewhat agreeing. In terms of the overall sample, only a small proportion was entrenched in their reluctance, with 6.63% reporting “strongly disagree” in both surveys. 1.67% of respondents who indicated willingness and 1.77% of respondents who indicated hesitancy in Wave 4 were subsequently vaccinated by Wave 5.

**Table 2:** Individual-level changes in willingness to receive a COVID-19 vaccine between Waves 4 and 5

	Willing to receive, Wave 5						
	Strongly Agree	Some-what Agree	Don’t Know	Some-what Disagree	Strongly Disagree	Already Vaccinated	Total
**Willing to receive, Wave 4**							
**Strongly Agree**	45.99	2.18	0.78	1.49	2.49	0.92	**53.85**
**Somewhat Agree**	9.05	4.37	0.38	1.25	1.68	0.25	**16.97**
**Don’t Know**	3.08	0.53	1.28	0.17	0.79	0.02	**5.88**
**Somewhat Disagree**	2.14	1.20	0.57	1.55	2.37	0.02	**7.85**
**Strongly Disagree**	4.61	1.58	0.40	1.75	6.63	0.47	**15.45**
**Total**	**64.87**	**9.86**	**3.41**	**6.21**	**13.96**	**1.69**	**100.00**

Note: Authors’ own calculations. Source: NIDS-CRAM Waves 4 and 5. Relevant estimates weighted using sampling weights. The sample was restricted to the balanced panel of respondents who were in both Wave 4 and Wave 5 (*n* = 4931)

**Table 3:** Individual-level distribution of willingness to receive a COVID-19 vaccine in Wave 5, based on willingness in Wave 4

	Willing to receive, Wave 5						
	Strongly Agree (64.87)	Some what Agree (9.86)	Don’t Know (3.41)	Some what Disagree (6.21)	Strongly Disagree (13.96)	Already Vaccinated (1.69)	Total
**Willing to receive, Wave 4**							
**Strongly Agree (53.85)**	85.40	4.05	1.46	2.76	4.61	1.72	**100.00**
**Some what Agree (16.97)**	53.30	25.74	2.22	7.36	9.88	1.50	**100.00**
**Don’t Know (5.88)**	52.39	9.07	21.80	2.89	13.45	0.40	**100.00**
**Some what Disagree (7.85)**	27.28	15.28	7.22	19.80	30.17	0.25	**100.00**
**Strongly Disagree (15.45)**	29.85	10.21	2.61	11.32	42.95	3.06	**100.00**

Note: Authors’ own calculations. Source: NIDS-CRAM Waves 4 and 5. Relevant estimates weighted using sampling weights. The sample was restricted to the balanced panel of respondents who were in both Wave 4 and Wave 5 (*n* = 4931). Values in headings indicate the proportion of the sample that provided each response option

### Demographic correlates of willingness to receive a COVID-19 vaccine in wave 5

Our aggregated estimates for vaccination intent among South African adults mask important variations across different groups. Additional file 3 reports results from a series of linear probability models that include demographic predictors of COVID-19 vaccine hesitancy in Wave 5. Respondents aged 18–24 years old were on average 8.5 percentage points more likely to be hesitant (*p* = .03). Those living in formal residential housing (*p* = .05) and those who reported trust in social media as a source of COVID-19 information (*p* = .01) were also significantly more likely to be hesitant. Respondents who self-reported their religion as Jewish (*p* < .01) and those who reported trust in community leaders as a source of COVID-19 information (*p* < .01) were significantly more likely to be willing to receive a vaccine, but these represented small shares of the overall sample. Household income quintile, household asset index quintile, social grant receipt status, recent household hunger were not significant predictors of vaccination intent.

We also estimated a linear probability model, reported in Additional file 4, that included the subset of participants who indicated that they were vaccine hesitant in Wave 4 and predicted their likelihood of reporting willingness to accept a vaccine in Wave 5. Hesitant respondents aged 18–24 were 5.6 percentage points more likely to shift to willingness in Wave 5 (*p* = .04). Those with a tertiary education (*p* < .01) and those with chronic conditions excluding overweight, obesity, and hypertension (*p* < .01) were significantly less likely to shift to willingness in Wave 5.

### Reasons for COVID-19 vaccine hesitancy

Among respondents in Wave 5 who did not “strongly agree” with the vaccine willingness question, 53.2% thought the vaccine was unsafe or harmful. Within this subgroup, more than half (52.2%) were very convinced of this, 17.0% were somewhat convinced, and 30.9% were only a little convinced. The subsample of respondents who said that they believed that the vaccine was unsafe (18% of the full sample) were asked why they believed this. Table 4 reports reasons that were cited by at least 2% of the subsample, as well as the strength of beliefs about vaccine safety concerns. At 32% of the subsample (6% of the full sample), the most frequently-cited reason was the belief that the vaccine testing was rushed. This was also the most strongly-held belief, with 65% of respondents being “very convinced” that the vaccine was unsafe. 20% of the subsample (4% of the full sample) cited concerns about side effects: 6% mentioned death, 4% mentioned blood clots, 1% mentioned HIV or cancer, 4% mentioned illness or other side effects, and 5% did not specify the side effect. Conspiracy theories were cited very infrequently; the most common of these were concerns about the vaccine being a government or global plot, with each mentioned by 6% of the subsample (1% of the full sample).

**Table 4:** Reasons for vaccine safety concerns and strength of beliefs (subsample of respondents who believed that the vaccine was unsafe)

Reason for concern about vaccine safety	% of subsample reporting reason	% of full sample reporting reason	% of subsample “very convinced” that the vaccine was unsafe
Vaccine testing rushed	32	6	65
Side effects	20	4	53
General safety concerns	12	2	61
Don’t trust	10	2	42
Vaccine government plot	6	1	54
Vaccine global plot	6	1	35
Ineffective	4	1	39
Vaccine will change DNA	4	1	52
Lack of information or knowledge	3	1	48
Vaccine fake for profit	2	< 1	51
Other	2	< 1	47
Wait and see	2	< 1	44

Note: Authors’ own calculations. Source: NIDS-CRAM Wave 5. Relevant estimates weighted using sampling weights

## Discussion

Our analysis of COVID-19 vaccination intent among adults in South Africa showed that 76% of those surveyed agreed with the statement that they would get a vaccine if one became available to them in April/May 2021, a statistically significant increase from 71% in February/March 2021. These estimates are consistent with results from other recent surveys conducted in South Africa [15]. The April/May survey’s interviews overlapped with the introduction of a large-scale rollout of vaccines to health workers, and we would expect that the overwhelmingly positive real-life vaccine experiences of health workers would prompt their friends, families and neighbours to reappraise fears based on hear-say rumours and conspiracies. On the other hand, the April/May survey also overlapped with the pause in vaccinations due to blood clotting concerns, which was expected to amplify worries about the safety of the vaccine. Encouragingly, the rise in vaccine acceptance shows that any negative influence of this pause in the vaccine rollout has not dominated attitudes towards vaccines. The greatest change between the two waves was in the proportion of respondents who indicated strong willingness to get a vaccine, at a 9 percentage point increase. Our findings on individual-level changes in vaccination intent between waves indicated that willingness to get a vaccine was more stable than reluctance to get a vaccine.

Our study has important limitations to note. Our use of a telephonic survey may have introduced sampling and non-response biases, though we attempted to mitigate them by having access to several phone numbers, including those of friends and family, for each respondent. We adjusted for systematic non-response through survey weights that drew on the 2017 NIDS survey, but factors that increased during the pandemic such as migration and challenges to mental health may have contributed to further non-response. Reporting bias due to social desirability bias may have affected our findings, though the degree of willingness to get a COVID-19 vaccine would likely have been overstated in this case. We are aware that our survey questions on vaccine hesitancy reflect stated intentions, and the literature has shown substantial gaps between stated intentions and realised decisions. We also acknowledge that slight methodological differences between the survey waves may limit their ability to be directly compared, though this is minimised with regards to the primary vaccine hesitancy outcome.

The implications of having one quarter of the adult population in South Africa expressing vaccine hesitancy require further attention. The community immunity threshold for SARS-CoV-2 is likely to be considerably higher in South Africa than the 60–70% that was previously estimated, due to the growing dominance of the highly transmissible Delta variant [16]. The main concern with the interpretation of an individual’s stated vaccine willingness is that intentions and attitudes do not always translate into behaviour [17]. This suggests that our finding of 76% willingness to get a vaccine may be an upper bound for the proportion that eventually accept it. Data on vaccine registrations support this perspective. We note that while 78% of those 60 years and over were willing to accept vaccines in April/May 2021, just over half of this age group had registered for vaccinations by July 4, two and a half months after registrations opened for this group [11]. This is consistent with international evidence that highlights that stated vaccine willingness is not always reflected in vaccine uptake [18, 19]. Proposed solutions to address barriers to registration and vaccination include offering these services at more convenient locations and times [19, 20]. For instance, the under-resourced province of Limpopo has partnered with community healthcare workers to conduct smartphone-enabled door-to-door registrations, and increased vaccination rates by administering doses on weekends [21].

Vaccination promotion campaigns should continue to frame vaccine acceptance as the norm. While it is important to address rumors and misinformation, frequently discussing vaccine scepticism can perversely give credence to myths by creating the impression that these beliefs are widespread and valid [22]. Our results indicate that people who are entrenched in opposition to the vaccine make up a significant minority of the population of South Africa. We also demonstrate that many strongly hesitant individuals change their minds over a short period of time. Aligned with what has been previously reported, [23] our study finds a significant association between trust in social media as a source of COVID-19 information and vaccine hesitancy.

Campaigns that promote vaccine acceptance should emphasize how many previously hesitant individuals have since become willing to get a vaccine. Our results add to the evidence emerging from other countries that younger adults are more reluctant to get vaccinated [15], but we also find that they are more likely to quickly change their mind. Approaches to reduce the tendency to rationalise vaccine refusal among this demographic may include framing decisions in terms of the prosocial impact of getting the vaccine, harnessing the desire to “return to normal” as a motivator, or emphasizing personal health benefits such as the lower likelihood of severe illness and hospitalisation [24, 25]. Finally, our results are consistent with prior findings that concerns about the vaccine’s safety, including those related to the roll-out timeline and possible side effects, are common reasons for hesitancy [15]. Vaccination campaigns should be consistent and transparent in communicating possible side effects, and can reframe beliefs by emphasising that minor side effects are a sign of the vaccine working [26].

## Conclusions

This study highlighted that the proportion of adults in South Africa willing to receive a COVID-19 vaccine increased between February/March and April/May 2021. This rise in vaccine acceptance suggests that setbacks in the vaccine rollout process, such as pauses due to safety concerns, have not dominated attitudes towards vaccines. We analysed a wide variety of information available in this dataset on respondents’ demographics, socioeconomic status, health status, and health beliefs. While most of these covariates did not significantly predict vaccination intent, we found that younger adults, those in formal housing, and those who trusted social media as a source of COVID-19 information were more likely to be hesitant about the vaccine. Though approximately one-quarter of the population were hesitant, a significant minority were entrenched in their reluctance between the two survey waves. Concerns about the vaccine’s development timeline and side effects were prevalent reasons for hesitancy. Vaccination promotion campaigns should emphasize that vaccine acceptance is the norm and employ strategies that account for the perceptions and attitudes of different demographic groups.

## Supplementary Information

**Additional file 1.** Vaccine-related questions in NIDS-CRAM. Survey questions related to the COVID-19 vaccine.

**Additional file 2.** Additional methods details. Additional details regarding the estimation of the proxies for socioeconomic status.

**Additional file 3.** Predictors of reluctance to receive a COVID-19 vaccine, NIDS-CRAM Wave 5. Linear probability model results for reluctance to receive a vaccine in Wave 5.

**Additional file 4.** Predictors of likelihood of vaccine-hesitant respondents in Wave 4 to indicate willingness to receive a vaccine in Wave 5. Linear probability model results for willingness to receive a vaccine in Wave 5, subset of respondents who indicated vaccine hesitancy in Wave 4.

**Additional file 5.**

## Data Availability

The manuscript’s data is available from the DataFirst website.

## Abbreviations

CI: Confidence interval
NIDS: The National Income Dynamics Study
NIDS-CRAM: The National Income Dynamics Study: Coronavirus Rapid Mobile Survey
