# Supplementary material for: Longitudinal changes in COVID-19 vaccination intent among South African adults: evidence from the NIDS-CRAM panel survey, February to May 2021
Source: BMC Public Health. 2022 Mar 2;22:422. doi: 10.1186/s12889-022-12826-5 (PMC8889513; doi:10.1186/s12889-022-12826-5)
Supplement: Supplementary file 1 — Additional file 1. Vaccine-related questions in NIDS-CRAM. Survey questions related to the COVID-19 vaccine. [file 12889_2022_12826_MOESM1_ESM.docx]

**Longitudinal changes in COVID-19 vaccination intent among South African adults: Evidence from the NIDS-CRAM panel survey, February to May 2021**

**ADDITIONAL FILE 1**

**Vaccine-related questions in NIDS-CRAM**

| **Question** | **Response Options** |
| --- | --- |
| Have you received a Coronavirus vaccine? | Yes |
|  | No |
|  | Refused |
|  | Don’t know |
| To what extent do you agree or disagree with the statement: If a vaccine for COVID-19 were available, I would get it?  **Interviewer: Read out options 1 – 4.** | Strongly agree |
|  | Somewhat agree |
|  | Somewhat disagree |
|  | Strongly disagree |
|  | Refused |
|  | Don’t know |
| Do you believe that the vaccine is unsafe or could harm you? | Yes |
|  | No |
|  | Refused |
|  | Don’t know |
| How convinced are you of this?  **Interviewer: Read out options 1 – 3.** | A little convinced |
|  | Somewhat convinced |
|  | Very convinced |
|  | Refused |
|  | Don’t know |
| Why do you believe that the vaccine is unsafe or harmful?  **Interviewer: Do not read out options, select all that apply.** | The vaccine is part of a government plot to harm/control me |
|  | The vaccine is part of a global plot to harm/control me |
|  | Vaccines are fake and are just being sold for profit |
|  | The vaccine contents will change my DNA |
|  | Vaccines can cause HIV or cancer |
|  | Vaccines go against my religious beliefs |
|  | Vaccine testing was rushed so we can’t be sure that they are safe. |
|  | COVID-19 isn’t real |
|  | Other (specify) |
|  | Refused |
|  | Don’t know |
