# Supplementary material for: Longitudinal changes in COVID-19 vaccination intent among South African adults: evidence from the NIDS-CRAM panel survey, February to May 2021
Source: BMC Public Health. 2022 Mar 2;22:422. doi: 10.1186/s12889-022-12826-5 (PMC8889513; doi:10.1186/s12889-022-12826-5)
Supplement: Supplementary file 2 — Additional file 2. Additional methods details. Additional details regarding the estimation of the proxies for socioeconomic status. [file 12889_2022_12826_MOESM2_ESM.docx]

**Longitudinal changes in COVID-19 vaccination intent among South African adults: Evidence from the NIDS-CRAM panel survey, February to May 2021**

**ADDITIONAL FILE 2**

**Additional Methods Details**

Due to concerns about the reliability and bias in the one-shot household income variable (26% of respondents did not report any value), we estimated two alternative proxies to capture differences in socioeconomic status for our sample. We formulated a deprivation and poverty index based on the respondent’s 2017 household assets and living circumstances. The list of household assets included ownership of a range of marketable assets, access to a savings account, to clean water, to electricity, and to adequate sanitation. Using multiple correspondence analysis, we extracted relative weights for each of these dimensions, and then compiled quintiles for the analysis.

We adjusted the household income variable to address potential bias in selection into bracket responses, the presence of outliers, and missing values. We did this in three steps. First, we reweighted observations by generating bracket weights to account for selection into bracket responses, as in Köhler and Bhorat and Hill and Köhler with the NIDS-CRAM Wave 2 data, as well as the Post-Apartheid Labour Market Series (PALMS) (1, 2). These weights were calculated as the inverse of the probability of an actual monetary (Rand) response in a particular bracket in a particular wave, multiplied by the sampling weight for each individual. In essence, this process weighted up individuals whose reported incomes were in brackets where the proportion of actual monetary responses is lower, relative to brackets where such response is high. Second, outlier values were identified and coded as missing by using the “extreme studentised residuals’’ approach as advised by Wittenberg (3). This was done by estimating a Mincerian-style Ordinary Least Squares (OLS) regression of the logarithm of nominal household income on a vector of observable covariates and identifying outliers as those observations with absolute residuals in excess of five. This process resulted in the household incomes of just three observations being coded as missing. Third, there is a wide range of methods available to impute values for observations with missing income data. Here, we simply imputed values by estimating and using the within-bracket median response. This process resulted in a significant reduction in the extent of missing values from 26.4% to 6.7% of the sample.

Additionally, we used responses about recent household hunger, and whether the respondent received a social grant, as alternative indicators of socioeconomic status. While we are aware of the shortcomings of each of these measures individually, we believe that collectively they cover an important share of the socioeconomic status variation of our sample of respondents.

**References**

1. Köhler T, Bhorat H. Social Assistance During South Africa's National Lockdown: Examining the COVID-19 grant, changes to the Child Support Grant. 2020. Available from: https://cramsurvey.org/wp-content/uploads/2020/09/9.-Ko%CC%88hler-T.-_-Bhorat-H.-2020-Social-assistanceduring-South-Africa%E2%80%99s-national-lockdown-Examining-the-COVID-19-grant-changes-tothe-Child-Support-Grant-and-post-October-policy-options.pdf.

2. Hill R, Köhler T. Mind the gap: analysing the effects of South Africa’s national lockdown on gender wage inequality. National Income Dynamics Study (NIDS)–Coronavirus Rapid Mobile Survey (CRAM) Wave. 2020;2. Available from: https://cramsurvey.org/wp-content/uploads/2020/09/7.-Hill-R.-_-Ko%CC%88hler-T.-2020-Mindthe-gap-Analysing-the-effects-of-South-Africa%E2%80%99s-national-lockdown-on-gender-wage-inequality.

3. Wittenberg M. Wages and wage inequality in South Africa 1994–2011: part 1–Wage measurement and trends. South African Journal of Economics. 2017;85(2):279-97.
