# Supplementary material for: Longitudinal changes in COVID-19 vaccination intent among South African adults: evidence from the NIDS-CRAM panel survey, February to May 2021
Source: BMC Public Health. 2022 Mar 2;22:422. doi: 10.1186/s12889-022-12826-5 (PMC8889513; doi:10.1186/s12889-022-12826-5)
Supplement: Supplementary file 3 — Additional file 3. Predictors of reluctance to receive a COVID-19 vaccine, NIDS-CRAM Wave 5. Linear probability model results for reluctance to receive a vaccine in Wave 5. [file 12889_2022_12826_MOESM3_ESM.docx]

**Longitudinal changes in COVID-19 vaccination intent among South African adults: Evidence from the NIDS-CRAM panel survey, February to May 2021**

**ADDITIONAL FILE 3**

**Table A1. Predictors of reluctance to receive a COVID-19 vaccine, NIDS-CRAM Wave 5**

| **Variable** | **Model 1**  **B (p)** | **Model 2**  **B (p)** | **Model 3**  **B (p)** | **Model 4**  **B (p)** |
| --- | --- | --- | --- | --- |
| **Gender**  [Ref. Male] |  |  |  |  |
| Female | 0.027 (0.23) | 0.023 (0.30) | 0.019 (0.40) | 0.006 (0.81) |
| **Age**  [Ref. 25-59] |  |  |  |  |
| 18-24 | 0.090* (0.01) | 0.086* (0.02) | 0.083* (0.02) | 0.085* (0.03) |
| 60+ | -0.051 (0.09) | -0.055 (0.07) | -0.069* (0.03) | -0.056 (0.10) |
| **Racial population group**  [Ref. African/Black] |  |  |  |  |
| Coloured | -0.065 (0.45) | -0.062 (0.47) | -0.065 (0.44) | -0.030 (0.71) |
| Indian/Asian | -0.120 (0.46) | -0.140 (0.40) | -0.140 (0.40) | -0.150 (0.39) |
| White | -0.003 (0.98) | -0.003 (0.97) | 0.003 (0.98) | 0.019 (0.82) |
| **Language**  [Ref. Zulu] |  |  |  |  |
| IsiNdebele | -0.150 (0.10) | -0.140 (0.12) | -0.130 (0.12) | -0.130 (0.13) |
| IsiXhosa | -0.021 (0.67) | -0.021 (0.66) | -0.019 (0.68) | -0.031 (0.52) |
| Sepedi | 0.071 (0.19) | 0.075 (0.16) | 0.076 (0.15) | 0.046 (0.43) |
| Sesotho | 0.038 (0.48) | 0.030 (0.57) | 0.026 (0.63) | 0.025 (0.64) |
| Setswana | -0.055 (0.34) | -0.063 (0.27) | -0.062 (0.28) | -0.055 (0.33) |
| SiSwati | 0.003 (0.97) | 0.013 (0.88) | 0.012 (0.88) | 0.026 (0.77) |
| Tshivenda | 0.220 (0.07) | 0.220 (0.06) | 0.210 (0.08) | 0.200 (0.09) |
| IsiTsonga | -0.010 (0.87) | -0.003 (0.96) | -0.009 (0.88) | -0.018 (0.72) |
| Afrikaans | 0.130 (0.15) | 0.120 (0.18) | 0.120 (0.19) | 0.090 (0.30) |
| English | 0.098 (0.26) | 0.097 (0.26) | 0.099 (0.25) | 0.090 (0.27) |
| Other | 0.780** (<0.01) | 0.820** (<0.01) | 0.840** (<0.01) | 0.820** (<0.01) |
| **Religion**  [Ref. Christian] |  |  |  |  |
| Not religious | 0.073 (0.10) | 0.072 (0.10) | 0.073 (0.09) | 0.082 (0.07) |
| Jewish | -0.190** (<0.01) | -0.180** (<0.01) | -0.180** (<0.01) | -0.160** (<0.01) |
| Muslim | 0.048 (0.74) | 0.058 (0.70) | 0.063 (0.67) | 0.061 (0.70) |
| Hindu | 0.030 (0.88) | 0.032 (0.86) | 0.033 (0.86) | 0.063 (0.75) |
| African Traditional | 0.040 (0.22) | 0.041 (0.21) | 0.041 (0.21) | 0.045 (0.18) |
| Other | 0.065 (0.47) | 0.069 (0.44) | 0.062 (0.48) | 0.070 (0.45) |
| **Importance of religion**  [Ref. Very unimportant] |  |  |  |  |
| Unimportant | -0.038 (0.64) | -0.034 (0.67) | -0.029 (0.72) | 0.032 (0.67) |
| Important | -0.014 (0.82) | -0.012 (0.85) | -0.013 (0.84) | 0.043 (0.47) |
| Very important | 0.010 (0.88) | 0.013 (0.84) | 0.014 (0.83) | 0.075 (0.22) |
| **Education**  [Ref. Completed secondary] |  |  |  |  |
| Up to primary | -0.015 (0.63) | -0.015 (0.65) | -0.019 (0.57) | -0.009 (0.80) |
| Up to secondary | 0.004 (0.89) | 0.004 (0.87) | 0.002 (0.94) | 0.014 (0.57) |
| Tertiary | -0.062* (0.04) | -0.065* (0.03) | -0.063* (0.04) | -0.035 (0.25) |
| **Residential area**  [Ref. Township] |  |  |  |  |
| Formal residential | 0.062* (0.03) | 0.062* (0.03) | 0.064* (0.03) | 0.060* (0.05) |
| Shack | 0.053 (0.12) | 0.059 (0.09) | 0.061 (0.08) | 0.064 (0.07) |
| Peri-urban | 0.004 (0.90) | 0.009 (0.77) | 0.011 (0.72) | 0.001 (0.98) |
| Traditional | -0.019 (0.58) | -0.013 (0.71) | -0.015 (0.67) | -0.022 (0.55) |
| Farm/small holding | -0.013 (0.75) | -0.008 (0.84) | -0.012 (0.76) | -0.018 (0.65) |
| **Infection risk**  [Ref. Not likely to get COVID-19] |  |  |  |  |
| Likely to get COVID-19 | -0.029 (0.13) | -0.03 (0.12) | -0.028 (0.14) | -0.024 (0.22) |
| Don’t know get COVID-19 | -0.024 (0.49) | -0.021 (0.56) | -0.023 (0.51) | -0.035 (0.33) |
| **Self-efficacy**  [Ref. Don’t think can avoid COVID-19] |  |  |  |  |
| Can avoid COVID-19 | -0.042 (0.20) | -0.042 (0.20) | -0.039 (0.23) | -0.054 (0.11) |
| Don’t know if can avoid COVID-19 | 0.047 (0.52) | 0.041 (0.57) | 0.045 (0.54) | 0.007 (0.93) |
| **Mortality risk**  [Ref. No reported health conditions] |  |  |  |  |
| Self-reported chronic conditions | -0.041 (0.06) | -0.041 (0.06) | -0.041 (0.06) | -0.039 (0.08) |
| Overweight | 0.008 (0.74) | 0.009 (0.72) | 0.009 (0.70) | 0.021 (0.40) |
| Obese | -0.013 (0.59) | -0.014 (0.55) | -0.011 (0.63) | -0.015 (0.53) |
| Hypertension | -0.032 (0.15) | -0.033 (0.12) | -0.035 (0.10 | -0.032 (0.15) |
| **Trusted information sources** |  |  |  |  |
| Social media | 0.110* (0.01) | 0.110* (0.01) | 0.100* (0.01) | 0.120* (0.01) |
| Community leader | -0.13** (<0.01) | -0.13** (<0.01) | -0.12** (<0.01) | -0.13** (<0.01) |
| **Deprivation and poverty index**  [Ref. Q1] |  |  |  |  |
| Q2 |  | 0.032 (0.41) | 0.029 (0.45) | 0.030 (0.45) |
| Q3 |  | -0.044 (0.18) | -0.044 (0.19) | -0.037 (0.30) |
| Q4 |  | -0.028 (0.47) | -0.029 (0.47) | -0.018 (0.67) |
| Q5 |  | 0.010 (0.79) | 0.011 (0.80) | 0.024 (0.58) |
| **Social grant**  [Ref. Respondent did not receive a grant] |  |  |  |  |
| Respondent received a grant |  |  | 0.043* (0.03) | 0.036 (0.08) |
| **Household hunger**  [Ref. No recent household hunger] |  |  |  |  |
| Recent household hunger |  |  | -0.038 (0.10) | -0.036 (0.14) |
| **Income**  [Ref. Q1] |  |  |  |  |
| Q2 |  |  |  | 0.010 (0.71) |
| Q3 |  |  |  | 0.013 (0.61) |
| Q4 |  |  |  | 0.016 (0.57) |
| Q5 |  |  |  | -0.024 (0.51) |
| **Intercept** | **0.35* (0.01)** | **0.36* (0.01)** | **0.35* (0.01)** | **0.27 (0.06)** |
| **Observations** | **4390** | **4390** | **4390** | **4390** |
| **R-squared** | **0.08** | **0.08** | **0.09** | **0.09** |

Notes: The regression also includes dummies for districts and a top-up sample, not reported here. Model 1 has no socioeconomic controls. Model 2 adds poverty quintiles as controls for socioeconomic status, Model 3 further expands the controls for socioeconomic status by adding grant receipt and recent household hunger and Model 4 is the most comprehensive, including also the adjusted income quintiles.

Source: NIDS-CRAM Wave 5, Wave 1 & NIDS 2017. Authors’ own calculations.
