# Supplementary material for: Longitudinal changes in COVID-19 vaccination intent among South African adults: evidence from the NIDS-CRAM panel survey, February to May 2021
Source: BMC Public Health. 2022 Mar 2;22:422. doi: 10.1186/s12889-022-12826-5 (PMC8889513; doi:10.1186/s12889-022-12826-5)
Supplement: Supplementary file 4 — Additional file 4. Predictors of likelihood of vaccine-hesitant respondents in Wave 4 to indicate willingness to receive a vaccine in Wave 5. Linear probability model results for willingness to receive a vaccine in Wave 5, subset of respondents who indicated vaccine hesitancy in Wave 4. [file 12889_2022_12826_MOESM4_ESM.docx]

**Longitudinal changes in COVID-19 vaccination intent among South African adults: Evidence from the NIDS-CRAM panel survey, February to May 2021**

**ADDITIONAL FILE 4**

**Table A2. Predictors of likelihood of vaccine-hesitant respondents in Wave 4 to indicate willingness to receive a vaccine in Wave 5**

| **Variable** | **B** | **p** |
| --- | --- | --- |
| **Gender**  [Ref. Male] |  |  |
| Female | 0.026 | 0.17 |
| **Age**  [Ref. 25-59] |  |  |
| 18-24 | 0.056* | 0.04 |
| 60+ | -0.006 | 0.83 |
| **Racial population group**  [Ref. African/Black] |  |  |
| Coloured | 0.047 | 0.70 |
| Indian/Asian | -0.079 | 0.52 |
| White | -0.068 | 0.52 |
| **Language**  [Ref. Zulu] |  |  |
| IsiNdebele | -0.002 | 0.96 |
| IsiXhosa | -0.018 | 0.51 |
| Sepedi | -0.048 | 0.07 |
| Sesotho | 0.011 | 0.77 |
| Setswana | 0.001 | 0.98 |
| SiSwati | 0.035 | 0.53 |
| Tshivenda | -0.084** | <0.01 |
| IsiTsonga | 0.025 | 0.58 |
| Afrikaans | 0.044 | 0.68 |
| English | 0.056 | 0.63 |
| Other | -0.21** | <0.01 |
| **Religion**  [Ref. Christian] |  |  |
| Not religious | 0.090* | 0.03 |
| Jewish | 0.044 | 0.62 |
| Muslim | 0.003 | 0.98 |
| Hindu | 0.180 | 0.39 |
| African Traditional | 0.026 | 0.31 |
| Other | -0.071* | 0.02 |
| **Importance of religion**  [Ref. Very unimportant] |  |  |
| Unimportant | -0.081 | 0.08 |
| Important | 0.031 | 0.52 |
| Very important | 0.016 | 0.73 |
| **Education**  [Ref. Completed secondary] |  |  |
| Up to primary | 0.026 | 0.40 |
| Up to secondary | -0.009 | 0.67 |
| Tertiary | -0.062** | <0.01 |
| **Residential area**  [Ref. Township] |  |  |
| Formal residential | -0.025 | 0.39 |
| Shack | -0.025 | 0.39 |
| Peri-urban | -0.035 | 0.18 |
| Traditional | -0.020 | 0.48 |
| Farm/small holding | 0.013 | 0.74 |
| **Infection risk**  [Ref. Not likely to get COVID-19] |  |  |
| Likely to get COVID-19 | 0.017 | 0.27 |
| Don’t know get COVID-19 | 0.056 | 0.14 |
| **Self-efficacy**  [Ref. Don’t think can avoid COVID-19] |  |  |
| Can avoid COVID-19 | -0.025 | 0.42 |
| Don’t know if can avoid COVID-19 | -0.042 | 0.48 |
| **Mortality risk**  [Ref. No reported health conditions] |  |  |
| Self-reported chronic conditions | -0.054** | <0.01 |
| Overweight | -0.001 | 0.98 |
| Obese | 0.005 | 0.81 |
| Hypertension | 0.007 | 0.69 |
| **Trusted information sources** |  |  |
| Social media | -0.048 | 0.12 |
| **Poverty and exclusion**  [Ref. Q1] |  |  |
| Q2 | -0.041 | 0.21 |
| Q3 | -0.029 | 0.36 |
| Q4 | -0.053 | 0.08 |
| Q5 | -0.028 | 0.41 |
| **Intercept** | **0.17*** | **0.01** |
| **Observations** | **3926** |  |
| **R-squared** | **0.05** |  |

Notes: Sceptics refer to those who in Wave 4 said they disagreed, somewhat disagreed or didn’t know whether they would accept vaccines if they were available. The regression also includes dummies for districts and a top-up sample, not reported here.

Source: NIDS-CRAM Wave 5, Wave 1 & NIDS 2017. Authors’ own calculations.
