# Supplementary material for: Longitudinal changes in COVID-19 vaccination intent among South African adults: evidence from the NIDS-CRAM panel survey, February to May 2021
Source: BMC Public Health. 2022 Mar 2;22:422. doi: 10.1186/s12889-022-12826-5 (PMC8889513; doi:10.1186/s12889-022-12826-5)
Supplement: Supplementary file 5 — Additional file 5. [file 12889_2022_12826_MOESM5_ESM.docx]

**Longitudinal changes in COVID-19 vaccination intent among South African adults: Evidence from the NIDS-CRAM panel survey, February to May 2021**

**ADDITIONAL FILE 5**

**Data Availability Statement**

The datasets generated and analysed during the current study are available in the DataFirst National Income Dynamics Study-Coronavirus Rapid Mobile Survey repository, <https://www.datafirst.uct.ac.za/dataportal/index.php/catalog/867> (Wave 4) and <https://www.datafirst.uct.ac.za/dataportal/index.php/catalog/873> (Wave 5).
